# YjcC, a c-di-GMP Phosphodiesterase Protein, Regulates the Oxidative Stress Response and Virulence of *Klebsiella pneumoniae* CG43

**DOI:** 10.1371/journal.pone.0066740

**Published:** 2013-07-23

**Authors:** Ching-Jou Huang, Zhe-Chong Wang, Hsi-Yuan Huang, Hsien-Da Huang, Hwei-Ling Peng

**Affiliations:** 1 Institute of Molecular Medicine and Biological Technology, National Chiao Tung University, Hsin Chu, Taiwan, Republic of China; 2 Department of Biological Science and Technology, National Chiao Tung University, Hsin Chu, Taiwan, Republic of China; 3 Institute of Bioinformatics and Systems Biology, National Chiao Tung University, Hsin Chu, Taiwan, Republic of China; Instituto de Biociencias - Universidade de São Paulo, Brazil

## Abstract

This study shows that the expression of *yjcC*, an in vivo expression (IVE) gene, and the stress response regulatory genes *soxR*, *soxS*, and *rpoS* are paraquat inducible in *Klebsiella pneumoniae* CG43. The deletion of *rpoS* or *soxRS* decreased *yjcC* expression, implying an RpoS- or SoxRS-dependent control. After paraquat or H_2_O_2_ treatment, the deletion of *yjcC* reduced bacterial survival. These effects could be complemented by introducing the Δ*yjcC* mutant with the YjcC-expression plasmid pJR1. The recombinant protein containing only the YjcC-EAL domain exhibited phosphodiesterase (PDE) activity; overexpression of *yjcC* has lower levels of cyclic di-GMP. The *yjcC* deletion mutant also exhibited increased reactive oxygen species (ROS) formation, oxidation damage, and oxidative stress scavenging activity. In addition, the *yjcC* deletion reduced capsular polysaccharide production in the bacteria, but increased the LD50 in mice, biofilm formation, and type 3 fimbriae major pilin MrkA production. Finally, a comparative transcriptome analysis showed 34 upregulated and 29 downregulated genes with the increased production of YjcC. The activated gene products include glutaredoxin I, thioredoxin, heat shock proteins, chaperone, and MrkHI, and proteins for energy metabolism (transporters, cell surface structure, and transcriptional regulation). In conclusion, the results of this study suggest that YjcC positively regulates the oxidative stress response and mouse virulence but negatively affects the biofilm formation and type 3 fimbriae expression by altering the c-di-GMP levels after receiving oxidative stress signaling inputs.

## Introduction

During infection, pathogens protect themselves from the oxidative burst of phagocytic cells and the challenging oxidative environments within cellular and extracellular compartments. Upon exposure to oxidative stress such as tellurite, paraquat or hydrogen peroxide, *E. coli* exhibits an increase in the intracellular ROS and the content of protein carbonyl groups [1]–[3]. Reactive oxygen species (ROS), including superoxide anion (O_2_^.−^), hydrogen peroxide (H_2_O_2_), and hydroxyl radicals (HO^.^), may damage DNA, proteins, and cell membranes and often lead to cell death [4], [5]. The bacterial defense mechanism includes sensing, avoiding, and removing the ROS [6]. In general, SodA, SodB, and SodC remove superoxide, whereas catalases (KatE and KatG) and peroxidases (AhpC and GST) remove hydrogen peroxide [7], [8]. These various stress defenses are controlled by regulators that respond to superoxide and redox-cycling drugs (e.g., SoxRS), hydrogen peroxide (e.g., OxyR), iron (e.g., Fur), or oxygen tension (e.g., FNR and ArcAB) [8]–[11]. Diguanylate cyclases (DGCs) and phosphodiesterases (PDEs) regulate the levels of bacterial second messenger cyclic di-GMP (c-di-GMP) by catalyzing molecular synthesis and hydrolysis, respectively [12], [13]. The regulatory roles of c-di-GMP appear in numerous bacteria in various cellular functions, including cell surface remodeling [14], cellulose synthesis [15], virulence [16], motility [17], and biofilm formation [18]–[20]. *E. coli* YfgF, which exhibits PDE activity, regulates not only surface cell remodeling but also the oxidative stress response by modulating c-di-GMP levels [11]. The disruption of *Salmonella enteric* Var. *typhimurium cdgR*, which encodes a PDE protein, also decreases bacterial resistance to hydrogen peroxide and accelerates death by macrophages [21].

*Klebsiella pneumoniae* pyogenic liver abscess isolates often carry heavy capsular polysaccharides (CPS) to avoid phagocytosis or death by serum factors [22], [23]. This thick and viscous structure also helps regulate the bacterial colonization and biofilm formation at the infection site [24]. Several regulators, such as RcsB, RmpA, RmpA2, KvhR, KvgA, and KvhA, help control the CPS biosynthesis by regulating the *cps* transcriptions in *K. pneumoniae* [25], [26]. An increase in CPS synthesis protects *K. pneumoniae* from oxidative stress [27]–[29]. However, whether the modulation of c-di-GMP affects CPS synthesis remains unclear.

The expression of *yjcC*, an IVE gene isolated from the liver abscess isolate *K. pneumoniae* CG43, is inducible in the presence of 10 µM paraquat [30]. Sequence analysis of YjcC shows a signal peptide followed by 2 transmembrane domains and a CSS motif at the N-terminal region, whereas the C-terminal contains a conserved EAL domain of the PDE enzyme [31]. In addition, the encoding gene *yjcC* is cluster-located with *soxRS* genes, suggesting that it plays a role in the oxidative stress response. This study investigates whether YjcC plays a role in oxidative stress defenses and if YjcC uses PDE activity to execute its regulation.

## Results

### The YjcC expression is paraquat inducible, and SoxRS and RpoS dependent

**Figure 1:**
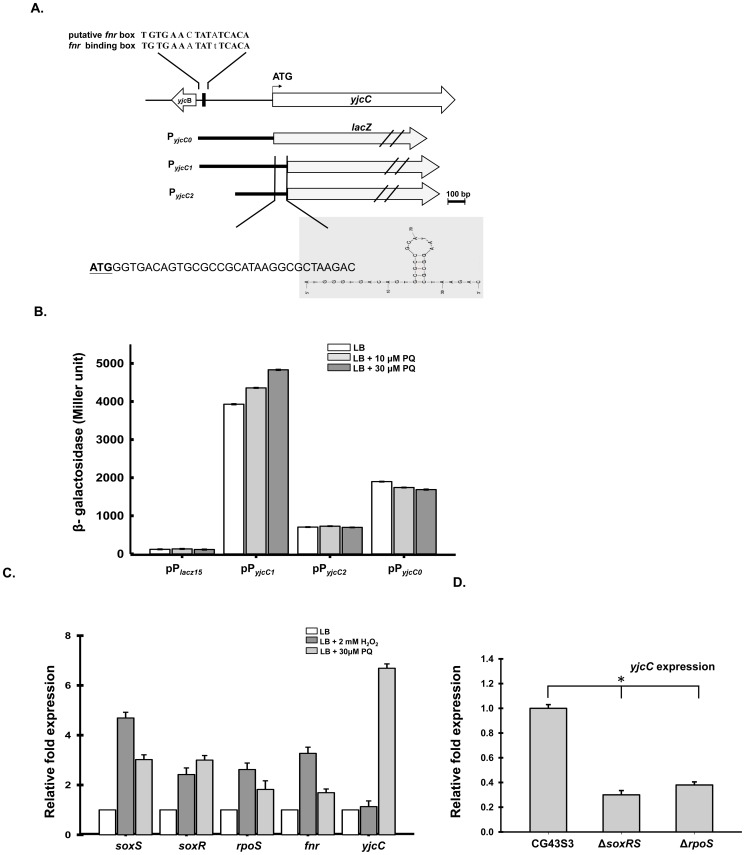
The *yjcC* is paraquat inducible, *and SoxRS and RpoS dependent*. (A) The putative promoters respectively containing 525 bp (P*_yjcC1_*), 385 bp (P*_yjcC2_*) and 415 bp (P*_yjcC0_*) of *yjcC* were isolated and cloned into the LacZ reporter plasmid placZ15 (22). (B) The recombinant plasmids placz15, pP*_yjcC1_*, pP*_yjcC2_* and pP*_yjcC0_* were then transformed to *K. pneumoniae* CG43Z01 and the β-galactosidase activities of the transformants grown to log-phase in LB broth were determined. The results are shown as an average of triplicate samples. Error bars indicate standard deviations. (C) Total RNA of *K. pneumoniae* CG43S3 was isolated after the bacteria were grown in 2 mM H_2_O_2_ or 30 µM of paraquat. Specific primer pairs used to detect the expression of *soxR*, *soxS*, *rpoS*, and *yjcC* are listed in Table S2. Relative fold expression was compared with the non-induced condition and determined by the 2^−ΔΔCt^ method (60). Error bars indicate standard deviation of the mean. Data are representative of three independent experiments. (D) The expression of *yjcC* was determined in Δ*soxRS* and Δ*rpoS* mutant by qRT-PCR. Data are representative of three independent experiments, *, P<0.001.

To confirm the previously reported paraquat-induced expression phenotype [30], the IVE DNA containing the 5′ non-coding region and part of the coding sequence of *yjcC* was isolated from *K. pneumoniae* CG43S3 and cloned in front of the promoterless *lacZ* gene of pLacZ15 [26]. The resulting plasmid was called pP*_yjcC1_*. The sequence analysis of P*_yjcC1_* shows a conserved Fnr box TGTGA-N_6_-TCACA [32] centered approximately 400-bp upstream of the *yjcC* start codon. This process also generated recombinant plasmids pP*_yjcC2_* and pP*_yjcC0_* carrying truncated forms of P*_yjcC1_*. These plasmids respectively removed the putative Fnr box and the small stem-loop sequence of the 33-bp coding region (Fig. 1A). As Fig. 1B shows, the bacteria containing pP*_yjcC1_* exhibited the highest level of β-galactosidase activity, whereas CG43S3[pP*_yjcC2_*] had the lowest activity. In addition, the activity of P*_yjcC1_*, but not P*_yjcC2_* nor P*_yjcC0_*, increased after added 10 µM paraquat to the culture medium. This paraquat-induced characteristic also appeared when the concentration increased to 30 µM, further enhancing the activity of P*_yjcC1_*.

As Fig. 1C shows, the addition 30 µM paraquat to the bacterial culture significantly increased the *yjcC* mRNA level. Compared to the expression of the well-characterized stress response regulators SoxS, SoxR, RpoS, and Fnr, the *yjcC* gene expression was more responsive to paraquat than to hydrogen peroxide exposure. This study also investigates whether *yjcC* is subjected to regulation by SoxRS or RpoS. As Fig. 1D shows, the deletion of *soxR*, *soxS*, or *rpoS* reduces the *yjcC* expression, implying that SoxRS and RpoS play a positive role in *yjcC* expression.

### YjcC plays a positive role in the oxidative stress response

**Figure 2:**
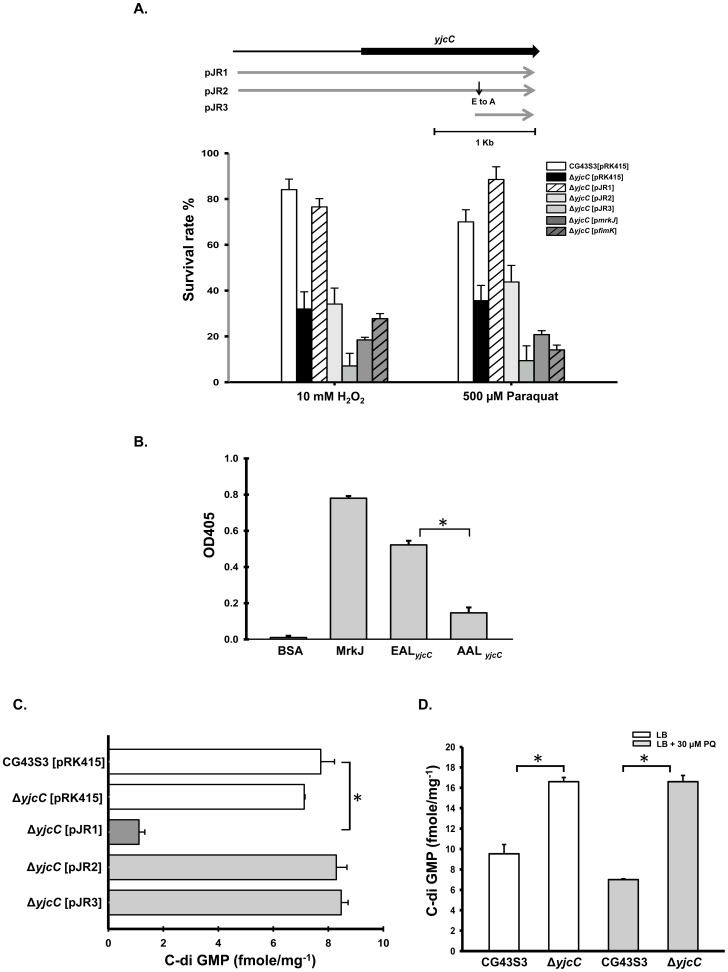
Analysis of the deletion effects of *yjcC* upon exposure to oxidative stress. Diagrammatic depiction of the YjcC complementation plasmids is shown in the upper panel. The plasmid pJR2 is identical to pJR1 except that the E residue was replaced with A by site directed mutagenesis. The plasmid pJR3 carries only the EAL domain region of YjcC. Overnight cultures were collected and refreshed grown in LB until OD600 reached 0.6–0.7. 500 µM paraquat or 10 mM H_2_O_2_ was then added and the cultures continued for 35 min and finally the cultures plated onto LB plates for colony formation. (B) Measurement of phosphodiesterase activity of the recombinant MrkJ protein (33), YjcC-EAL domain and YjcC-AAL domain proteins. BSA was used as a negative control. The activity is demonstrated using bis (pNpp) as substrate by the release of p-nitrophenol. (C), (D) Quantification of the c-di-GMP levels using the ELISA kit according to the manual (Wuhan EIAab Science Co., Ltd). Three independent experiments were performed for the measurement Error bars shown are standard deviations, and asterisks indicate the differences with a statistical significance, P<0.001.

Paraquat is a superoxide anion generator. Thus, the paraquat-inducible expression suggests that YjcC plays a role in the oxidative stress response. To investigate this possibility, an *yjcC* deletion mutant was generated through an allelic exchange strategy. As Fig. 2A shows, the *yjcC* deletion mutant was more sensitive to paraquat and hydrogen peroxide when compared to the wild type bacteria *K. pneumoniae* CG43S3. The deletion effect could be complemented by transforming the *yjcC* expression plasmid pJR1 into the mutant. However, introducing the mutant pJR2, which expresses the mutant form of YjcC with the conserved E residue of the EAL domain replaced by A or pJR3 (which carries the coding region of the YjcC EAL domain), had no complementation effect. Neither of the two EAL-domain protein encoding plasmids p*mrkJ* and p*fimK*, which carry PDE activity, could complement the *yjcC* deletion effect. These results suggest that the stress response is YjcC dependent and both the N-terminal signaling receiving region and the EAL domain of YjcC are required and specific for an oxidative stress response.

To determine if the YjcC-EAL domain exhibits PDE activity, the recombinant expression plasmid containing the DNA coding for the EAL domain of YjcC or the AAL coding region of pJR2 was constructed and overexpresed in *E. coli*, and the recombinant proteins were purified. Figure 2B shows that the purified EAL domain protein exhibits PDE activity towards pNpp. This activity is lower than the level of the recombinant MrkJ [33], but considerably higher than the activity of the recombinant protein AAL*_yjcC_*. As Fig. 2C shows, the c-di-GMP level of CG43S3Δ*yjcC*[pJR1] was significantly lower than those of CG43S3[pRK415], CG43S3Δ*yjcC*[pJR2], or CG43S3Δ*yjcC*[pJR3]. This suggests that YjcC in vivo functions as a PDE enzyme capable of reducing the intracellular c-di-GMP levels. The deletion of *yjcC* gene from CG43S3 increased the c-di-GMP amounts and the difference between the levels was much more apparent after the bacteria exposure to 30 µM paraquat (Fig. 2D). This also suggests that YjcC is able to degrade c-di-GMP and the catalytic activity could be enhanced by oxidative stress.

### Deletion of yjcC places bacteria in an oxidative stress state

**Figure 3:**
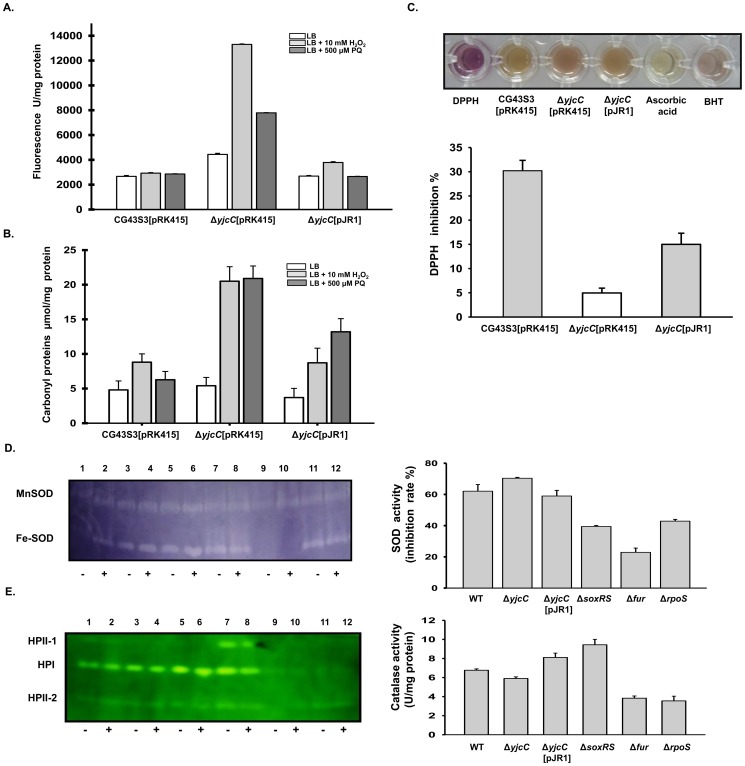
Deletion of *yjcC* places bacteria in an oxidative stress state. (A) Cytoplasmic ROS content determination. (B) The oxidation determination of the cytoplasmic proteins and membrane lipids. The log-phased bacteria *K. pneumoniae* CG43S3[pRK415], Δ*yjcC*[pRK415] and Δ*yjcC*[pJR1] were exposed to 500 µM paraquat or 10 mM H_2_O_2_ for 40 min and the intracellular peroxide levels and the carbonyl groups were determined. Bars represent standard deviations (n = 4). (C) The DPPH radical scavenging activity measurement. The upper panel shows that, correlating with the antioxidant activity, the color of DPPH gradually changes from purple to yellow. Ascorbic acid and Butylated hydroxytoluene (BHT) were used as positive control. The lower panel shows quantitative measurement of the DPPH scavenging activity of the log-phased bacteria *K. pneumoniae* CG43S3[pRK415], Δ*yjcC*[pRK415] and Δ*yjcC*[pJR1]. Bars represent standard deviations (n = 4). (D) SOD and (E) catalase activity determination as described in Materials and Methods. Left panels, in gel staining for the activity of Mn-SOD and Fe-SOD (D) and catalase HPI and HPIIs (E); Lanes 1, 2: CG43S3; 3, 4: Δ*yjcC*; 5, 6: Δ*yjcC*[pRK415-pJR1]; 7, 8: Δ*soxRS*; 9, 10: Δ*fur*; 11, 12: Δ*rpoS*. Lanes 1, 3, 5, 7, 9, and 11 are protein extracts of the bacteria with no stress treatment; 2, 4, 6, 8, and 10 are protein extracts of the bacteria with paraquat. Right panels, quantitative measurement of the total SOD (D) and catalase activity (E).

As Figs. 3A and 3B show, the deletion of y*jcC* after treatment of H_2_O_2_ or paraquat significantly raised the levels of the fluorescent probe H2DCFDA (used to monitor the formation of ROS) and carbonyl proteins. The introduction of pJR1 into CG43S3Δ*yjcC* mutant appeared to reduce the levels of ROS and the carbonyl proteins, showing that YjcC is involved in the removal of ROS or damaged molecules. Thus, this study also investigates the anti-oxidant activity of YjcC. As Fig. 3C shows, the deletion of *yjcC* reduced the oxidant scavenging activity, as assessed by the absorbance change at 517 nm for the decolorization degree of the purple color, supporting the possibility that YjcC modulates anti-oxidant activity in a certain manner. Numerous studies have shown that Fur and RpoS affect and regulate numerous SODs and catalases [7], [34]–[37]. Figure 3D shows that zymogel analysis and total activity measurement exhibit significant changes in the SOD or catalase activity after the deletion of *fur* or *rpoS*. However, the deletion of *yjcC* has no apparent influence on SOD or catalase activity, suggesting that the YjcC-dependent anti-oxidant enzyme remains to be identified.

### YjcC plays a regulatory role in the virulence, CPS production, biofilm formation, and type 3 fimbriae expression

**Figure 4:**
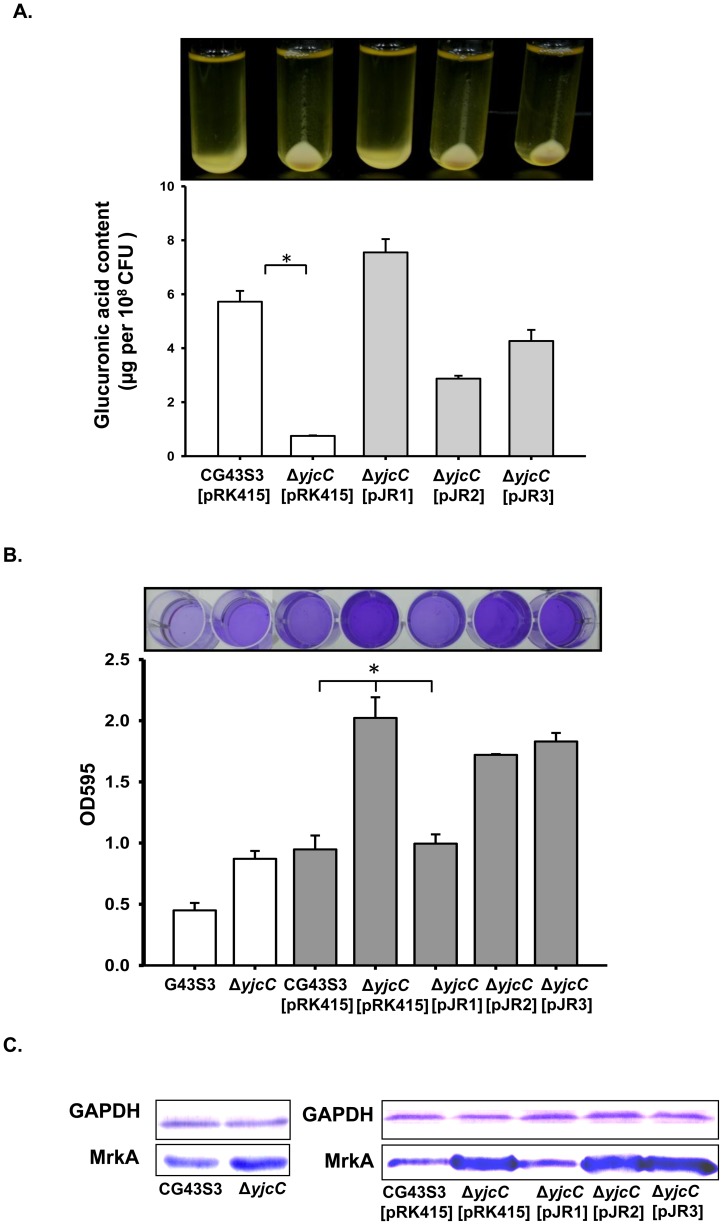
YjcC affects the CPS biosynthesis, biofilm formation and MrkA production. (A) Sedimentation analysis (upper panel) and glucuronic acid content measurement (lower panel). Bacteria were grown overnight in LB broth at 37°C and subjected to centrifugation at 4000 rpm for 5 min. The glucuronic acid contents are expressed as the average of the triplicate samples. Error bars indicate standard deviations. *, *P*<0.001 compared to the parental strain CG43S3 (*n* ≧ 3). (B) Biofilm formation as assessed using crystal violet staining (upper panel) and spectrometry (lower panel). Bacteria were grown at 37°C in polystyrene plates for 24 h, the sessile bacteria stained with crystal violet, and then the stained cells eluted with 95% ethanol. *, *p*<0.001. Data are representative of three independent experiments (triplicate in each experiment). (C) Western blotting analysis for the expression of MrkA. Bacteria were grown overnight at 37°C with agitation in LB broth, and then total proteins extracted for western blot analysis. GAPDH was probed as a protein loading control.

YjcC, previously identified as an IVE gene product, is likely involved in infection [30]. To investigate whether YjcC is a virulence factor for the bacteria to establish infection, a mouse peritonitis model was employed. As Table 1 shows, the LD50 to Balb/c mice increased approximately 10-fold after *yjcC* deletion; introducing Δ*yjcC* with pJR1, but not pJR2 or pJR3, could restore the LD50. This indicates that YjcC expression at a certain stage is required for mouse infection. It is interesting to note that the Δ*yjcC* colony is smaller and less mucoid, as determined by a string test [25], than its parental strain on LB agar plate. Therefore, sedimentation analysis and glucuronic acid content measurement are carried out to determine the CPS production. As Fig. 4A shows the deletion of *yjcC* reduces CPS production. The CPS deficient phenotype can be fully complemented with the transformation of pJR1 into the Δ*yjcC* mutant. However, transforming the mutant with pJR2 or pJR3 partially restores glucuronic acid production.

**Table 1:** YjcC effect on the mouse virulence.

Strain	LD50
CG43S3	1×10^4^
CG43S3Δ*yjcC*	7.8×10^5^
CG43S3Δ*yjcC* [pJR1]	3.3×10^4^
CG43S3Δ*yjcC* [pJR2]	1.1×10^6^

The tested bacterial strains CG43S3, CG43S3Δ*yjcC*,CG43S3Δ*yjcC*[pJR1] and CG43S3Δ*yjcC*[pJR2] were cultured in LB medium at 37°C for overnight.

Five mice of a group were injected intraperitoneally with bacteria resuspended in 0.2 ml of saline in 10-fold steps graded doses. The LD_50_, based on the number of survivors after one week, were calculated by the method of Reed and Muench (53) and expressed as CFU.

The second messenger c-di-GMP plays an important role in bacterial biofilm formation [14]–[16]. Figure 4B shows that the biofilm formation activity of Δ*yjcC* appears to increase compared to the parental strain, whereas that transformed with pJR1 decreases biofilm formation. This can be attributed to the level changes of c-di-GMP (Fig. 2C), which indicates that the YjcC expression of pJR1 significantly reduces the c-di-GMP level, thus reducing biofilm forming activity. Moreover, type 3 fimbriae is a major determinant of biofilm formation in *K. pneumoniae* [38]. Therefore, this study also investigates the deletion effect on type 3 fimbriae expression. As Fig. 4C shows, the western blot hybridization with anti-MrkA antibody shows that the *yjcC* deletion also increased the major pilin MrkA production of type 3 fimbriae.

### Effects of YjcC overexpression assessed using a transcriptome study

This study uses comparative transcriptome analysis between CG43S3[pRK415] and CG43S3[pJR1] to gain further insights into how YjcC executes its regulation. Analysis of the genome annotation of liver abscess isolate *K. pneumoniae* NTHU-K2044 [39] shows that the increased expression of *yjcC* significantly enhances the expression of 34 genes. As Table 2 shows, the YjcC-activated genes can be categorized into 12 functional groups. These include the oxidative stress response genes *grxA*, *ybbN*, *dinI*, *priB*, and *stpA*, which are involved in anti-oxidation [40], [41] or DNA repair [41], the heat shock chaperone protein encoding genes *ibpB*, *ibpA*, *htpG*, and *dnaK*, which are generally induced in stress conditions [42], and the genes coding for chaperone ClpB and PspB to protect protein from aggregation and help maintain proton motive force (PMF) to counteract stress conditions [43], [44]. Increasing the expression of *yjcC* also enhanced the expression of PilZ domain protein MrkH and the LuxR-type transcription factor MrkI. Conversely, 29 genes whose expressions were significantly repressed by the increase of YjcC expression include *fumB*, which is regulated by *fnr* under limited oxygen and anaerobic conditions [45]. Other YjcC negatively affected genes are metabolite transporter genes and genes coding for permease and energy metabolism involved in the synthesis of amino acids (Table 3).

**Table 2:** Significantly upregulated genes by *yjcC* overexpression.

Proposed function	Gene name	Fold^a^ expression	ORF^b^ ID
**Oxidative response/repair/sos responsive**			
Glutaredoxin I	*grxA*	2.6	KP1_1843
Thioredoxin	*ybbN*	2.4	KP1_1349
DNA damage inducible protein I	*dinI*	2.1	KP1_2061
**Heat shock response/chaperones/protein modification**			
Heat shock protein	*ibpB*	5.9	KP1_5467
Heat shock protein	*ibpA*	5.6	KP1_2622
Heat shock protein 90	*htpG*	2.9	KP1_1331
Heat shock protein 70	*dnaK*	3.5	KP1_0835
Protein disaggregation chaperon	*clpB*	3.2	KP1_4170
**Phage shock protein B**	*pspB*	2.1	KP1_2344
**Ci-di GMP metabolite**			
EAL containing protein	*yjcC*	6.3	KP1_0324
PilZ domain protein	*mrkH*	3.2	KP1_4551
**Energy/intermediary metabolism**			
Carbamoyl phosphate synthase	*carA*	3.2	KP1_0853
Ascorbate specific PTS family enzyme		3.1	KP1_2793
D-arabinitol dehydrogenase		2.9	KP1_3760
Allulose-6-phosphate 3-epimerase		2.3	KP1_2791
Xylulokinase		2	KP1_3759
**Transporter**			
ABC transporter ATP-binding protein		2.5	KP1_5267
Putative transport protein/kinase	*ydjN*	2	KP1_2272
**Amino acid biosynthesis**			
Sulfate adenylate transferase subunit 2	*cysD*	2.4	KP1_4384
**Nucleotide biosynthesis and metabolism**			
Methionine sulfoxide reductase A	*msrA*	2.5	KP1_0489
**DNA replication/recombination/repair**			
Primosomal replication protein N	*priB*	2.1	KP1_0469
DNA binding protein, nucleoid associated	*stpA*	2.6	KP1_4260
**Cell surface structures**			
Prepillin peptidase dependent protein		2.6	KP1_0311
Two component system connector	*ycgZ*	2.6	KP1_1728
Pullulanase-specific type II secretion system outer membrane lipoprotein	*pspD*	2.3	KP1_0998
**Regulators**			
LysR family transcriptional regulator		2.8	KP1_4352
Transcriptional activator	*nhaR*	2.1	KP1_0838
LuxR family regulatory protein	*mrkI*	2.6	KP1_4552
Hemolysin modulating protein	*hha*	2.6	KP1_1317
Transcriptional antiterminator of glycerol uptake operon		2.1	KP1_1112
DNA binding transcriptional activator	*pspC*	2.1	KP1_2344
**Hypothetical proteins**			
ParB family protein HP		3.3	KP1_2152
formylglycine generating sulfatase		2.6	KP1_3378
Membrane anchored protein		2	KP1_2896

**Table2 and 3.** Significantly upregulated and downregulated genes by YjcC overexpression. The selected genes show more than 2 log_2_ fold change (in absolute value) in the abundance of transcript between *K. pneumoniae* CG43S3[pRK415] and CG43S3[pJR1].

a Log_2_ fold change represents the log_2_ ratio of mRNA transcript levels of CG43S3[pJR1] to CG43S3[pRK415].

b Open reading frame (ORF) ID is as annotated from *K. pneumoniae* NTUH K2044.

**Table 3:** Significantly downregulated genes by *yjcC o*verexpression.

Proposed function	Gene name	Fold^a^ expression	ORF^b^ ID
**Anaerobic response protein**			
Anaerobic class I fumarate hydratase	*fumB*	−5.7	KP1_2562
**Transporter**			
Alanine/serine/glycine transport protein		−6.8	KP1_2505
ABC transport system ATP binding protein		−4.5	KP1_3173
PTS transporter subunits II ABC		−5.0	KP1_3804
Methylgalactoside transporter inner membrane component	*mglC*	−4.8	KP1_0277
Methylgalactoside transporter systemSubstrate-binding component	*mglB*	−4.2	KP1_3815
Putative ABC transport system component		−4.6	KP1_3175
Molybdate ABC transporter system		−4.4	KP1_3995
Maltose/maltodextrin transporter ATP binding protein	*malK*	−4.2	KP1_0276
Sugar ABC transport system permease component		−4.1	KP1_1424
Putative ABC transporter	*mocB*	−3.7	KP1_1423
Putative rhizopine uptake ABC transporter	*proY*	−3.7	KP1_1422
**Permease**			
Putative PTS permease		−6.5	KP1_0760
Putative amino acid permease		−4.3	KP1_1204
**Amino acid biosynthesis**			
Arginine succinyltransferase		−4.5	KP1_2499
Acetylornithine transaminase		−5.0	KP1_2498
Putative glutamine synthetase		−3.3	KP1_2006
**Energy/intermediary metabolism**			
Succinate antiporter		−6.4	KP1_2563
Succinylarginine dihydrolase		−5.3	KP1_2502
Glucosamine-fructose-6-phosphate aminotransferase		−6.0	KP1_0764
NADH: flavin oxidoreductase		−5.8	KP1_2565
Phospho-beta glucosidase		−5.2	KP1_3803
Acety-CoA synthetase	*asc*	−4.3	KP1_0342
Succinylglutamate desuccinylase	*astE*	−4.0	KP1_2256
Phosphomannosemutase	*manB*	−3.4	KP1_3702
Putative monooxygenase subunit		−3.1	KP1_1996
Putative acid phosphotase		−3.1	KP1_3725
**Regulators**			
DNA binding transcriptional repressor	*lldR*	−4.5	KP1_5297
**Cell surface structures**			
Maltoporin	*lamB*	−4.8	KP1_0277

a Log_2_ fold change represents the log_2_ ratio of mRNA transcript levels of CG43S3[pJR1] to CG43S3[pRK415].

b Open reading frame (ORF) ID is as annotated from *K. pneumoniae* NTUH K2044.

**Figure 5:**
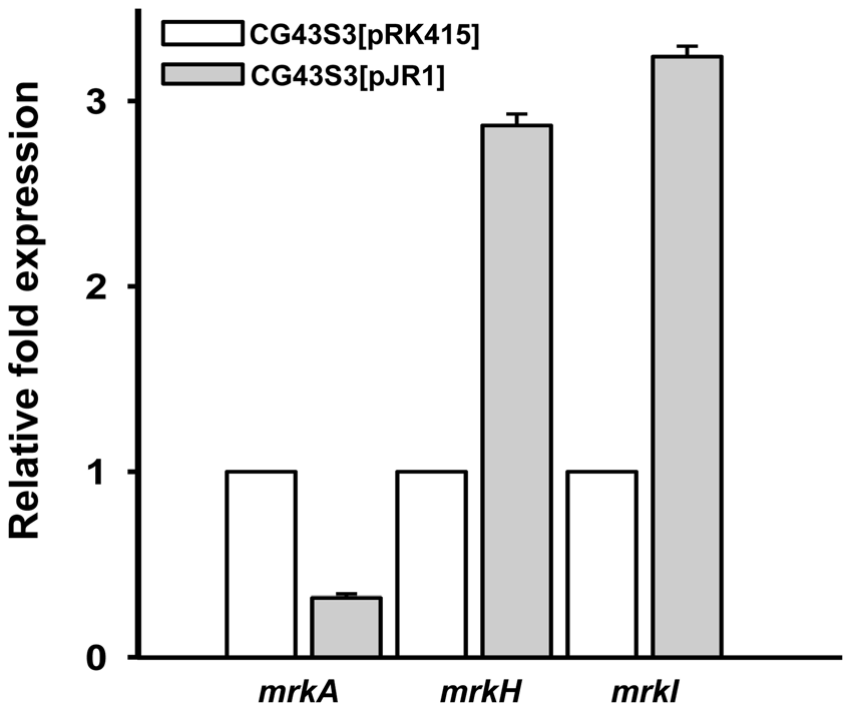
qRT-PCR analysis of the expression of *mrkA*, *mrkH*, and *mrkI*. Total RNA of *K. pneumoniae* CG43S3[pRK415] and CG43S3[pJR1] were isolated after the bacteria were grown overnight in LB supplemented with 12.5 µg/ml tetracycline. Specific primer pairs used to detect the expression of *mrkA*, *mrkH*, and *mrkJ* are listed in Table S2. Relative fold expression was compared with the non-induced condition and determined by the 2^−ΔΔCt^ method (60). Error bars indicate standard deviation of the mean. Data are representative of three independent experiments.

As assessed by qRT-PCR analysis, Fig. 5 shows that the mRNA level of *mrkH* and *mrkI*, respectively increased 2.87- and 3.24-fold in CG43S3[pJR1] compared to that of CG43S3[pRK415]. In contrast, the *mrkA* transcript levels dropped to approximately 1/3 of that of CG43S3[pRK415].

## Discussion

In *E. coli*, all the described genes inducible by paraquat are a part of the SoxRS regulon [46]–[48]. The expression of *yjcC* is RpoS-dependent in *E. coli* [49]. Consistent with this finding, the *yjcC* expression in *K. pneumoniae* is affected by RpoS and by SoxRS at the transcriptional level. No SoxRS or RpoS binding box appears within the putative promoter sequence, suggesting the possibility of an indirect control. In *E. coli*, the FNR regulator controls the transition between aerobic and anaerobic growth at the transcriptional level [50]. The conserved *fnr* binding box present in the upstream non-coding region of *yjcC* implies an FNR dependent control of YjcC expression. Thus, the *yjcC* regulation by FNR likely occurs in poorly oxygenated environments.

The *K. pneumoniae* NTUH K2044 genome contains 27 genes encoding GGDEF-, EAL-, and GGDEF-EAL-domain proteins of potential DGC and PDE enzyme activity [51]. The *yjcC* encoding gene is also identified as member of the protein family. The family regulation specificity is determined by the sensory domain of the DGC or PDE proteins. Figure 2A shows that the PDE expression plasmids p*fimK* and p*mrkJ*, which contain the respective coding region with putative promoter, failed to complement *yjcC* deletion. This may be due to *fimK* and *mrkJ* genes are not induced in comparison to *yjcC* in the presence of paraquat. There is also the possibility that the N-terminal peptide of approximately 300 aa of YjcC plays a role in the oxidative stress response besides signal sensing. Various sensory domains can bind to small molecular signals, and through this connection, modulate the levels of c-di-GMP [52], [53]. Although the signal for the CSS-motif remains unknown, we speculate that YjcC-mediated signaling sensing may occur in the periplasmic space because of the signal peptide and the transmembrane domain at the N-terminal region. The purified recombinant EAL domain of YjcC exhibits PDE activity. However, the amount of c-di-GMP in CG43S3Δ*yjcC*[pJR3] is approximately the same as that in CG43S3Δ*yjcC*[pRK415] and CG43S3Δ*yjcC*[pJR2], suggesting that the EAL domain has extremely low levels of PDE activity. The N terminal region of YjcC likely requires receiving some signal from outside, and the interaction of the sensory domain with signaling molecules activates the PDE activity. This is supported by Fig. 2D showing that the deletion of *yjcC* gene from CG43S3 raised the c-di-GMP levels and the influence was much more apparent after the bacteria exposure to 30 µM paraquat.

The deletion of *yjcC* decreases the CPS production and the virulence attenuation, indicating that YjcC is required to avoid the damage of oxidative stress. In bacteria, superoxide dismutase and catalase are common antioxidant enzymes that scavenge ROS from oxidative stress. The redox proteins (including GrxA and YbbN) required for maintaining redox status in bacteria are also protect bacteria from oxidative stress [54], [55]. The deletion of *yjcC* has no apparent effect on the SOD and catalase activity, but appears to increase the transcription of *grxA* and *ybbN*. This suggests that YjcC is involved in regulating the redox levels in bacteria after oxidative stress. The chaperone protein ClpB stabilizes protein and suppresses the protein aggregation induced by heat or other stresses [43], [56]. The phage shock proteins, PspB and PspC, act as positive regulators to transduce stress signal(s) to PspA through protein–protein interaction, maintaining the proton-motive force under extracytoplasmic stress conditions [44]. Transcriptome analysis shows that an increase in YjcC induces the expression of several heat shock proteins and chaperones, suggesting that YjcC is involved in regulating anti-stress responses. The fumarase gene *fumB*, which is expressed under anaerobic cell growth conditions, is regulated by Fnr and ArcA [57], [58]. The result of *yjcC* expression reduced the *fumB* transcripts implies that YjcC is a component of the Fnr and ArcA regulatory pathway.

In *K. pneumoniae*, the second messenger c-di-GMP activates type 3 fimbriae expression through MrkHI activation [18]. However, the increased synthesis of *mrkH* and *mrkI* transcripts by the overexpression of *yjcC* remains unclear. We have found that *mrkHI* expression is barely detected under LB culture (unpublished observation). Under oxidative stress pressure, the N-terminal region of YjcC may turn on the expression of MrkHI. Thus, the N-terminal region likely plays a determinant role in YjcC-dependent regulation. *E. coli* YdeH has c-di-GMP cyclase activity [59]. The transcriptome analysis as shown in Table S3 revealed that the transcript levels of *mrkA*, *mrkH*, and *mrkI* in CG43[pRK415-*ydeH*], with c-di-GMP level of 23.1 fmole/mg^−1^ (data not shown), significantly increased compared to those of CG43S3[pJR1]. This also indicates that *mrkHI* expression is c-di-GMP level-dependent and the N-terminal part of YjcC plays a positive regulatory role in the expression of *mrkHI*.

Overall, these results indicate that the YjcC-mediated regulatory system is considerably more complex than expected. During infection, the transition from aerobic to microaerobic conditions or the transition from a microaerobic to oxidative stress environment, YjcC may be activated through sensory regulatory systems on the N-terminal region. Thereafter, YjcC modulates the levels of c-di-GMP to affect the expression of the downstream regulatory pathways. In conclusion, YjcC regulates the oxidative stress response, mouse virulence, CPS synthesis, biofilm formation, and type 3 fimbriae expression. This most likely occurs through the adjustment of c-di-GMP levels after receiving outside signals.

## Materials and Methods

### Ethics Statement

All animal experiments were performed in strict accordance with the recommendation in the Guide for the Care and Use of Laboratory Animals of the National Laboratory Animal Center (Taiwan), and the protocol was approved by the Animal Experimental Center of National Chiao Tung University (Permit number: 00990006). All surgery was performed under anesthesia, and all efforts were made to minimize suffering.

### Plasmids, bacterial strain, and growth conditions

Table S1 presents the bacterial strains and plasmids used in this study. *E. coli* and *K. pneumoniae* CG43 [25], [26] and its derivatives were propagated at 37°C in Luria-Bertani (LB) broth. The antibiotics used include ampicillin (100 µg/mL), chloramphenicol (35 µg/mL), kanamycin (25 µg/mL), streptomycin (500 µg/mL), and tetracycline (12.5 µg/mL). Table S2 presents the primers used in this study.

### Measurement of promoter activity

The promoter reporter plasmids were individually mobilized from *E.coli* S17-1λ *pir* to *K. pneumoniae* strains by conjugation. The β-galactosidase activity was measured as described previously [26]. The bacteria were grown to the log phase in the LB medium (OD600 of 0.6–0.7) and 100 µL of the culture was mixed with 900 µL of Z buffer (60 mM Na2HPO4, 40 mM NaH2PO4, 10 mM KCl, 1 mM MgSO4, and 50 mM β-mercaptoethanol), 17 µL of 0.1% sodium dodecyl sulfate (SDS), and 35 µL of chloroform, followed by vigorous shaking. After incubation at 30°C for 10 min, 200 µL of a 4-mg/mL concentration of o-nitrophenyl-β-D galactopyranoside (ONPG; Sigma-Aldrich, Milwaukee, WI) was added to the mixture to initiate the reaction. When yellow coloration appeared, the reaction was stopped by adding 500 µL of 1 M Na2CO3 to the mixture. The absorbance at OD420 was recorded, and the activity was expressed as Miller units. Each sample was assayed in triplicate, and at least 3 independent experiments were conducted. The data shown were calculated from one representative experiment, and are presented as the means and standard deviations from triplicate samples.

### Real-time PCR analysis

Total RNA was isolated from bacteria using High Pure RNA isolation Kit (Roche, Germany), and the residual DNA was eliminated with RNase-free DNase I (Roche, Germany). The cDNAs used for PCR were synthesized from 1.5 µg RNA using random hexamer primer from RevertAid™ H Minus First strand cDNA synthesis Kit (Fermentas, Canada). An ABI Prism 7000 Detection system was used to perform PCR following the manufacturer instructions, and the products were detected using SYBR Green RCR Master Mix (Roche, Germany). The RNA samples were normalized to the level of 23S rRNA. PCR analysis was performed in triplicate in a reaction volume of 25 µL containing 12.5 µL of SYBR Green PCR Master Mix, 300 nM of primer pairs, 9.5 µL of distilled H_2_O, and 1 µL of cDNA. Samples were heated for 10 min at 95°C and amplified for 40 cycles for 15 s at 95°C and 60 s at 60°C. Quantification was performed using the 2^−ΔΔCt^ method [60].

### Construction of the gene deletion mutants and complementation plasmids

Specific gene deletion was introduced into *K. pneumoniae* CG43 using an allelic-exchange strategy as previously described [26]. Two DNA fragments of approximately 1000-bp flanking both sides of the deleted region were cloned into pKAS46 [25]. The plasmid pKAS46 is a suicide vector containing *rpsL*, which allows positive selection with streptomycin for vector loss. The resulting plasmids were mobilized from *E.coli* S17-1λ *pir* to *K. pneumoniae* CG43S3 or CG43S3Δ*lacZ*, by conjugation, respectively. The transconjugants, with the plasmid integrated into the chromosome through homologous recombination, were selected with ampicillin and kanamycin on M9 agar plates. Several of the colonies were grown overnight in LB broth at 37°C and then spread onto an LB agar plate containing 500 µg of streptomycin/mL. Streptomycin-resistant and kanamycin-sensitive colonies were selected, and the deletion was verified by PCR. Table S1 presents the resulting mutant strains. To obtain the complementation plasmids, DNA fragments containing the *yjcC*, and *soxRS* loci were PCR amplified using the primer pairs pjr1-F/pjr1-R, pjr2-F/pjr2-R, and pjr3-F/pjr3-R (Table S2). The PCR products were cloned in pRK415 [61] to generate pJR1(pRK415-*yjcC*), pJR2(pRK415-*yjcC*-AAL), pJR3(pRK415-EAL domain only of *yjcC*), p*mrkJ*, and p*fimK* respectively (Table S1).

### Site-directed mutagenesis

Site-directed mutagenesis was performed on the plasmid pJR1 to substitute the critical residue E with A in the EAL domain of YjcC using a QuickChange site-directed mutagenesis kit and following the manufacturer protocols (Stratagene). The resulting PCR product contained one point mutation, corresponding to the E303-to-A303 change in the active EAL site. The resulting PCR product was digested with *Bam*HI and *Hin*dIII and ligated into *Bam*HI/*Hin*dIII-digested plasmid pRK415-pJR2.

### Paraquat and H2O2 survival assessment

One hundred microliter of bacteria grown overnight were inoculated in LB and incubated at 37°C to OD600 of 0.6–0.7. An aliquot of the bacteria was collected by centrifugation and then resuspended in 500 µM of paraquat and 10 mM H_2_O_2_ respectively, and then subjected to 37°C incubation for 35 min. The colony-forming unit (CFU) of the bacteria was counted after the stress treatment, and the survival rate was determined by the CFU ratio. This study presents the representative data of at least 3 independent experiments. Every sample was assayed in triplicate, and this study presents the average activity and standard deviation.

### Cloning, expression, and purification of the recombinant proteins

The coding regions of the EAL or AAL domains of *yjcC* were PCR amplified with the primer sets yEAL-F/yEAL-R and yAAL-F/yAAL-R (Table S2) and cloned in the NdeI/XhoI site in pET30b (Novagen, Madison, WI). This process generated pET30b-EAL or pET30b-AAL with a carboxyl-terminus His tag (RcsB-His6). The resulting plasmids pEAL (pET30b-EAL of *yjcC*) and pAAL (pET30b-AAL of *yjcC*) were individually transformed into *E. coli* BL21(DE3)/pLysS (Invitrogen), and the overproduction of the recombinant protein was induced by adding 0.5 mM IPTG for 4 h at 37°C. The recombinant proteins were then purified from the soluble fraction of the total cell lysate by affinity chromatography using His-Bind resin (Novagen). The purified proteins were then dialyzed against 1 TBS (Tris-buffered saline; pH 7.4) containing 10% glycerol at 4°C overnight, followed by condensation with PEG 20000. The purity was determined by SDS-PAGE.

### Phosphodiesterase activity assay

The recombinant plasmids pET30b-EAL*_yjcC_*, pET30b-AAL*_yjcC_*, and pET30b-*mrkJ* were transformed into *E. coli* BL21(DE3), and the protein induced expression using 0.5 mM IPTG at 22°C for 12 h. The PDE activity was determined using the synthetic chromogenic substrate *bis*(*p*-nitrophenyl) phosphate (bis-pNPP) (Sigma-Aldrich) as previously described [62], [63]. The specific PDE activity was determined using purified proteins and by measuring the release of *p*-nitrophenol (*p*NP) at 405 nm. The calculations in this study use an extinction coefficient of 1.78*104/M*cm for *p*-nitrophenol. Control BSA without extracts was included to account for any non-enzymatic bis-pNPP hydrolysis and MrkJ, which carries PDE activity as a positive control [33].

### In vivo ci-di GMP content

To measure the c-di-GMP contents, cellular extracts were prepared as described [64]. The cultured bacteria were collected and treated with formaldehyde (0.19% final concentration) and then pelleted by centrifugation. The pellet was suspended in water and heated to 95°C for 10 min before the nucleotides were extracted by 65% ethanol. The lyophilized samples were then resuspended in water and this suspension was used for c-di-GMP detection with a cyclic diguanylate ELISA kit (Wuhan EIAab Science co., Ltd). The ci-di GMP activity of crude extracts (1 mg of total protein/mL) containing WT-pRK415 vector only, pJR1, and pJR2 in Δ*yjcC* was also assayed as described above.

### Determination of intracellular ROS concentration

Bacterial cultures were grown exponentially (OD600 = 0.6–0.7) and exposed to 10 mM of hydrogen peroxide or 500 µM of paraquat for 40 min. Cells were centrifuged at 13000 *g*, washed with 10 mM potassium phosphate (pH 7.0) buffer (Buffer A), and suspended in 500 µL of the same buffer, which contained 10 µM 2′,7′-dihydrodichlorofluorescein diacetate (H2DCFDA). After shaking for defined periods in darkness at room temperature, cells were centrifuged as mentioned and washed twice with 500 µL of Buffer A. Cells were suspended in 500 µL of Buffer A and disrupted by sonication. After centrifugation at 13000 *g*, aliquots of 100 µL supernatants were used to determine fluorescence intensity (excitation 490 nm and emission 519 nm) as described [1], [2].

### Oxidation of cytoplasmic proteins

Bacteria was grown to an OD600 0.6–0.7 in the presence of 10 mM hydrogen peroxide. After incubating for 30 min at 37°C, crude extracts were prepared and suspended in 500 µL of Buffer A and then disrupted by sonication. After centrifugation at 13000 *g*, 4 aliquots of 10 mM dinitrophenylhydrazine (DNPH) were added to the supernatant and the mixture was incubated at room temperature for 1 h with occasional stirring. Proteins were precipitated by adding one volume of 20% trichloroacetic acid (TCA) and centrifuged at 13000 *g* for 5 min. The precipitate was washed 3 times with a mixture of ethanol: ethyl acetate (1∶1). Finally, the sediment was dissolved in 450 µL of 6M guanidine hydrochloride/dithiothreitol and carbonyl concentration was determined spectrophotometrically at 370 nm (e = 22000 M-1 cm-1) [2], [3].

### Evaluation of antioxidant activity with DPPH assay

DPPH radical scavenging activity was estimated using the method of Liyana-Pathirana and Adedapo [65], [66]. When DPPH accepts an electron donated by an antioxidant compound, the free radical is decolorized from a stable purple color to yellow which can be quantitatively measured from the changes in absorbance. A solution of 0.135 mM DPPH in methanol was prepared, and 1.0 mL of this solution was mixed with 1.0 mL of extract in methanol containing 0.02–0.1 mg of the extract. The reaction mixture was thoroughly vortexed and left in the dark at room temperature for 30 min. The absorbance of the mixture was measured with a spectrophotometer at 517 nm, with ascorbic acid and BHT as references. The scavenge ability to remove DPPH radicals was calculated using the following equation: DPPH radical scavenging activity (%) = [(Abs control−Abs sample)]/(Abs control)]×100, where Abs control is the absorbance of DPPH radical+methanol, and Abs sample is the absorbance of DPPH radical+sample extract/standard.

### Determination of SOD and catalase enzyme activity

**C**ell-free extracts were harvested (15000 rpm, 4°C, 20 min) from the exponential phased bacteria (OD_600_ = 0.7–0.8) and suspended in ice-cold potassium phosphate buffer (50 mM, pH 7). After cells were disrupted by ultra-sonication, cell debris was removed by 12000 rpm centrifugation for 10 min at 4°C, and the supernatant was collected. The extraction of total proteins was conducted on ice and the concentrations were estimated using the Bradford method [67]. Aliquots of the extracted proteins were individually loaded onto 10% native polyacrylamide gels and the proteins were separated at a constant voltage of 150 V for 2 h. The gels were then removed and stained for SOD and CAT activity using the methods of Beauchamp and Fridovich [68] and Woodbury et al. [69], respectively. The SODs were localized by soaking gels in 2.45 mM nitro blue tetrazolium for 20 min, followed by immersion in a solution of 50 mM phosphate buffer (pH 7.0), 0.028 mM riboflavin, and 0.028 M TEMED (*N*,*N*,*N*_,*N*_-tetramethylethylenediamine). The gels were then removed from the solution and exposed to light for approximately 20 min. The SOD activity produced achromatic zones in the purple gel. The expression of CAT activity was identified by soaking gels in 10 mM hydrogen peroxide for 30 min with gentle shaking and then transferring the gels to a solution of 1% ferric chloride and 1% potassium ferricyanide for 10 min. The localized-CAT produced colorless bands on a dark green background.

The SOD activity was determined using a spectrophotometer at 25°C using the xanthine oxidase–cytochrome C method [68]. The assay mixture in 0.7 mL contained 50 mM potassium phosphate (pH 7.8), 0.1 mM EDTA, 50 mM xanthine, 1.7 mU xanthine oxidase, and 10 mM cytochrome C. The reduction of cytochrome C was measured at A550. One unit (U) of SOD activity was defined as the amount of enzyme required to inhibit the reduction rate of cytochrome C by 50%. Catalase activity was also determined spectrophotometrically at 25°C by monitoring the decrease in A240 in 50 mM Tris/HCl buffer (pH 8.0) [1]. One unit (U) of activity was defined as the amount of enzyme that catalyses the oxidation of 1 mmol H_2_O_2_ min^−1^ under assay conditions.

### Mouse lethality assay

The bacterial virulence in mice was determined as described [25]. Female BALB/c mice (aged 4 to 5 wk) were obtained from the National Laboratory Animal Center and acclimatized in an animal house for 7 d. The tested bacterial strains were cultured overnight in LB medium at 37°C. Four mice in each group were injected intraperitoneally with 0.2 mL of bacterial suspension in saline in 10-fold graded doses. The LD50 values were calculated using the Reed and Muench method [70] based on the number of survivors after 14 d.

### Extraction and quantification of capsular polysaccharides

The bacterial CPS was extracted using the method described [25], [71]. A 500 µL sample of overnight-grown bacteria was mixed with 100 µL of 1% Zwittergent 3–14 (Sigma-Aldrich, Milwaukee, WI) in 100 mM citric acid (pH 2.0) and then incubated at 50°C for 20 min. After centrifugation, a 250 µL sample of the supernatant was transferred to a new tube, and the CPS was precipitated with 1 mL of absolute ethanol. The pellet was dried and dissolved in 200 µL of distilled water, and 1200 µL of 12.5 mM borax in H2SO4 was then added. The mixture was vigorously mixed, boiled for 5 min, and cooled before adding 20 µL of 0.15% 3-hydroxydiphenol (Sigma-Aldrich, Milwaukee, WI). The absorbance at 520 nm was measured, and the uronic acid content was determined from a standard curve of glucuronic acid and expressed as micrograms per 10^8^ or 10^9^ CFU.

### Biofilm formation assay

The ability of bacteria to form biofilm was analyzed as described, with a minor modification. Bacteria were diluted 1/100 in LB broth supplemented with the appropriate antibiotics, and this dilution was inoculated into each well of a 96-well micro titre dish (Orange Scientific) and statically incubated at 37°C for 48 h. Planktonic cells were removed, and the wells were washed once with distilled water to remove unattached cells. Crystal violet (0.1% w/v; Sigma) was used to stain the attached cells for 30 min. Unattached dye was rinsed by washing 3 times with distilled water, and the stained biomass was solubilized in 1% (w/v) SDS. The absorbance was determined at 595 nm, and relative bacterial biofilm-forming activities were observed.

### Western blot analysis

*K. pneumoniae* CG43S3 and its derived mutants were grown in LB broth with agitation at 37°C. The bacterial total protein, approximately 5 µg per lane, was then subjected to western blot analysis using MrkA antiserum. Aliquots of total cellular lysates were resolved by SDS-PAGE, and the proteins were electrophoretically transferred onto a polyvinylidene difluoride (PVDF) membrane (Millipore, Billerica, MA, USA). After incubation with 5% skim milk at room temperature for 1 h, the membrane was washed 3 times with 1× PBS. The membrane was subsequently incubated with diluted anti-GAPDH (GeneTex Inc.) and anti-MrkA serum at room temperature for 1 h. After 3 washes with 1× PBS, a 5000-fold diluted alkaline phosphatase-conjugated anti-rabbit immunoglobulin G was added and incubated for 1 h. The blot was washed again and the bound antibodies were detected using the chromogenic reagents BCIP (5-bromo-4-chloro-3-indolyl phosphate) and NBT (Nitro blue tetrazolium).

### Transcriptome analysis

Total RNA from CG43S3[pRK415] and CG43S3[pJR1] were isolated using Trizol reagent (Invitrogen), subsequently purified using RNeasy MinElute Cleanup Kit (Qiagen), and eluted in RNase-free water. The RNA samples were sequenced on Illumina's sequencing instrument. In the data analysis pipeline, we used the FASTX-Toolkit to remove or trim deep sequencing reads containing low quality bases or adaptor sequences. To estimate expression levels of genes, all the remaining reads were mapped to the *Klebsiella pneumoniae* NTUH-K2044 genome [39]using TopHat and determined using Cufflinks package. The genes were identified as significantly transcript abundance changed in CG43S3[pRK415] as compared to that in CG43S3[pJR1] if the log_2_ fold change was greater than 2 (up and down). Finally, functional annotation tools, such as DAVID, can be used to illustrate the biological regulation role from Gene Ontology or KEGG pathway database.

## Supporting Information

Table S1 **Strains and plasmids used in this study.**
(TIF)

Table S2 **Oligonucleotide primers used in this study.**
(TIF)

Table S3 **RNA sequencing analysis of expression of** ***mrkA*** **,** ***mrkH*** **and** ***mrkJ*** **.**
(TIF)
