# Supplementary figures and images for: YjcC, a c-di-GMP Phosphodiesterase Protein, Regulates the Oxidative Stress Response and Virulence of Klebsiella pneumoniae CG43
Source: PLoS One. 2013 Jul 23;8(7):e66740. doi: 10.1371/journal.pone.0066740 (PMC3720812; doi:10.1371/journal.pone.0066740)

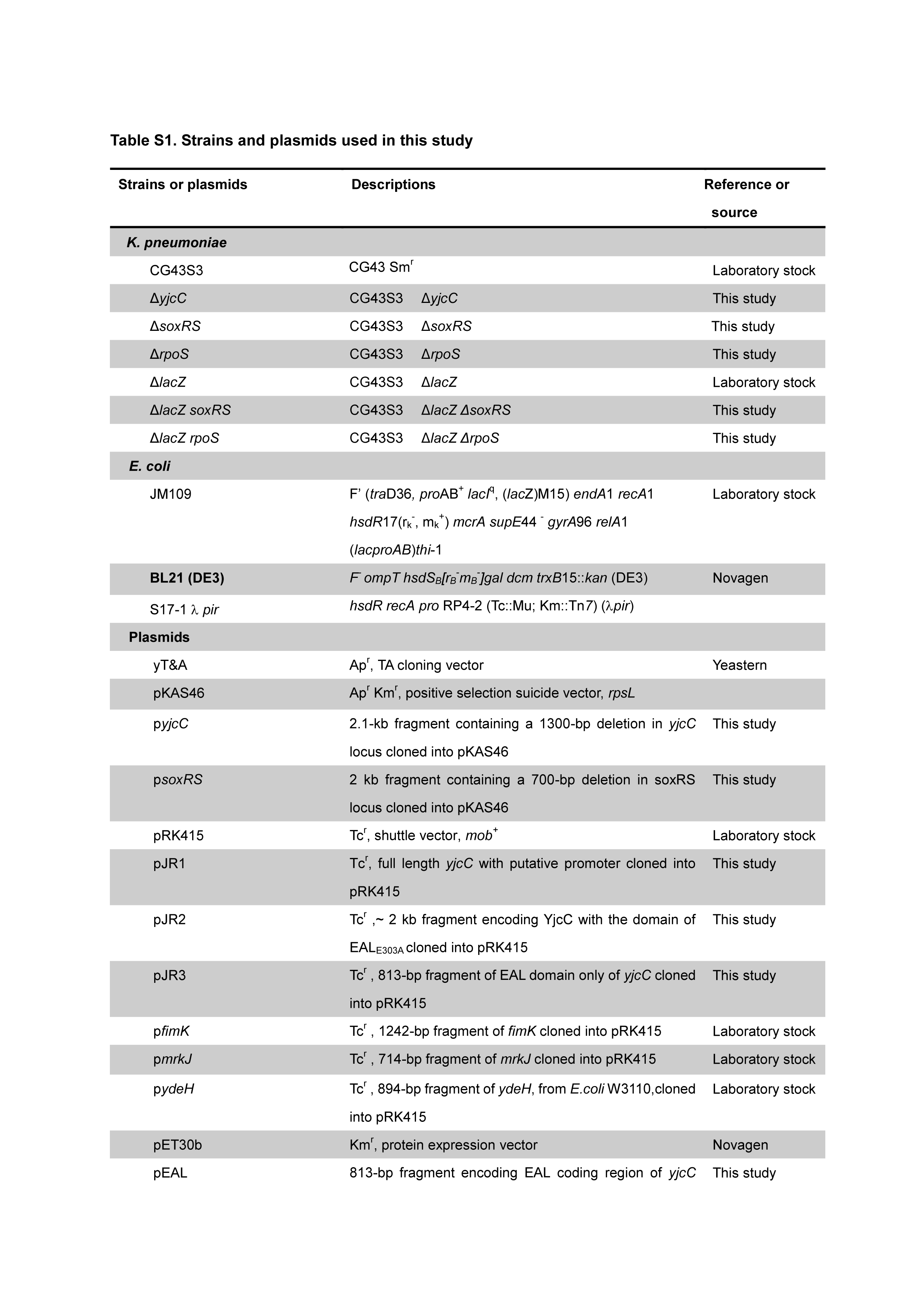

Supplement: Table S1 — Strains and plasmids used in this study. (TIF) [file pone.0066740.s001.tif]

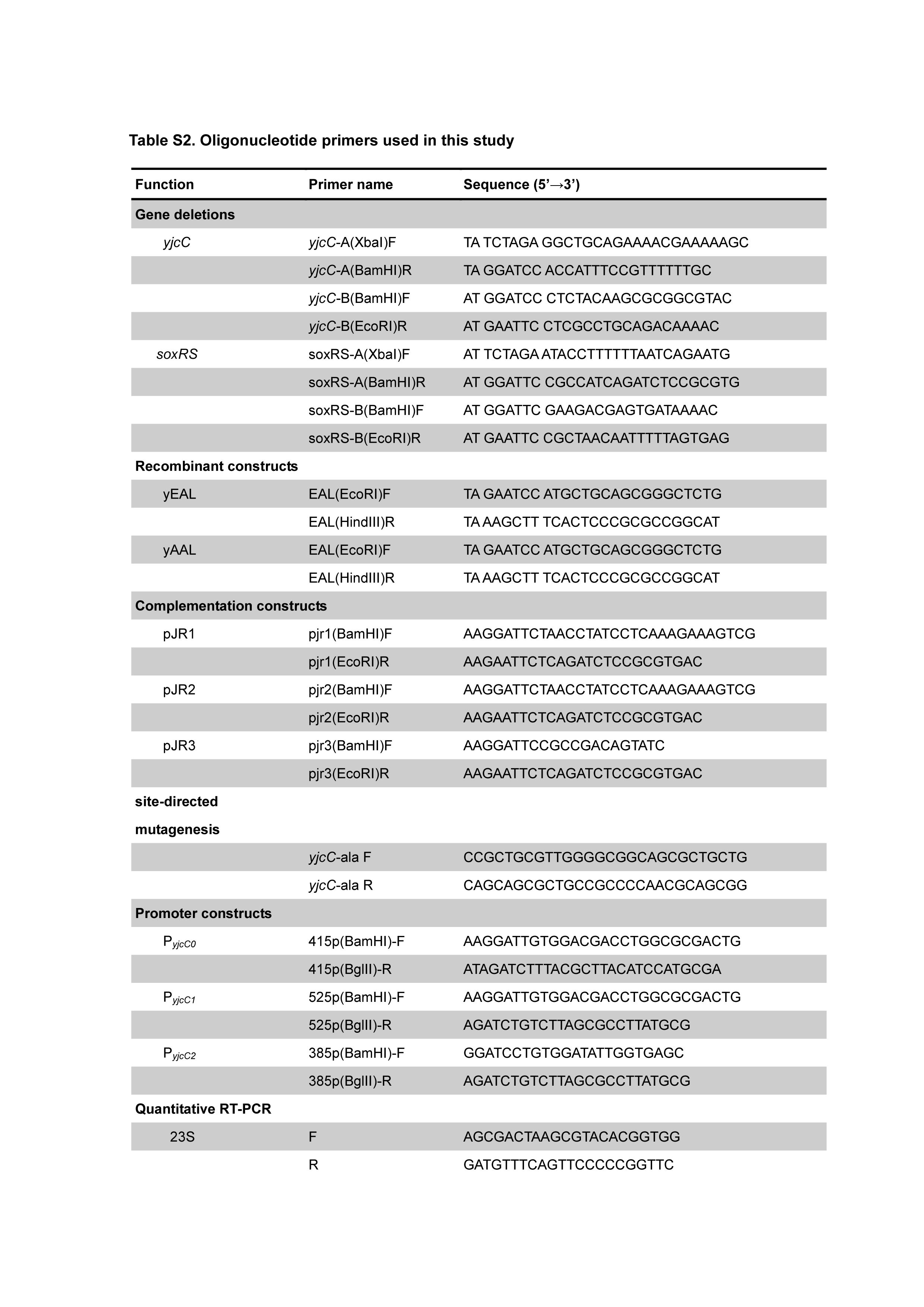

Supplement: Table S2 — Oligonucleotide primers used in this study. (TIF) [file pone.0066740.s002.tif]

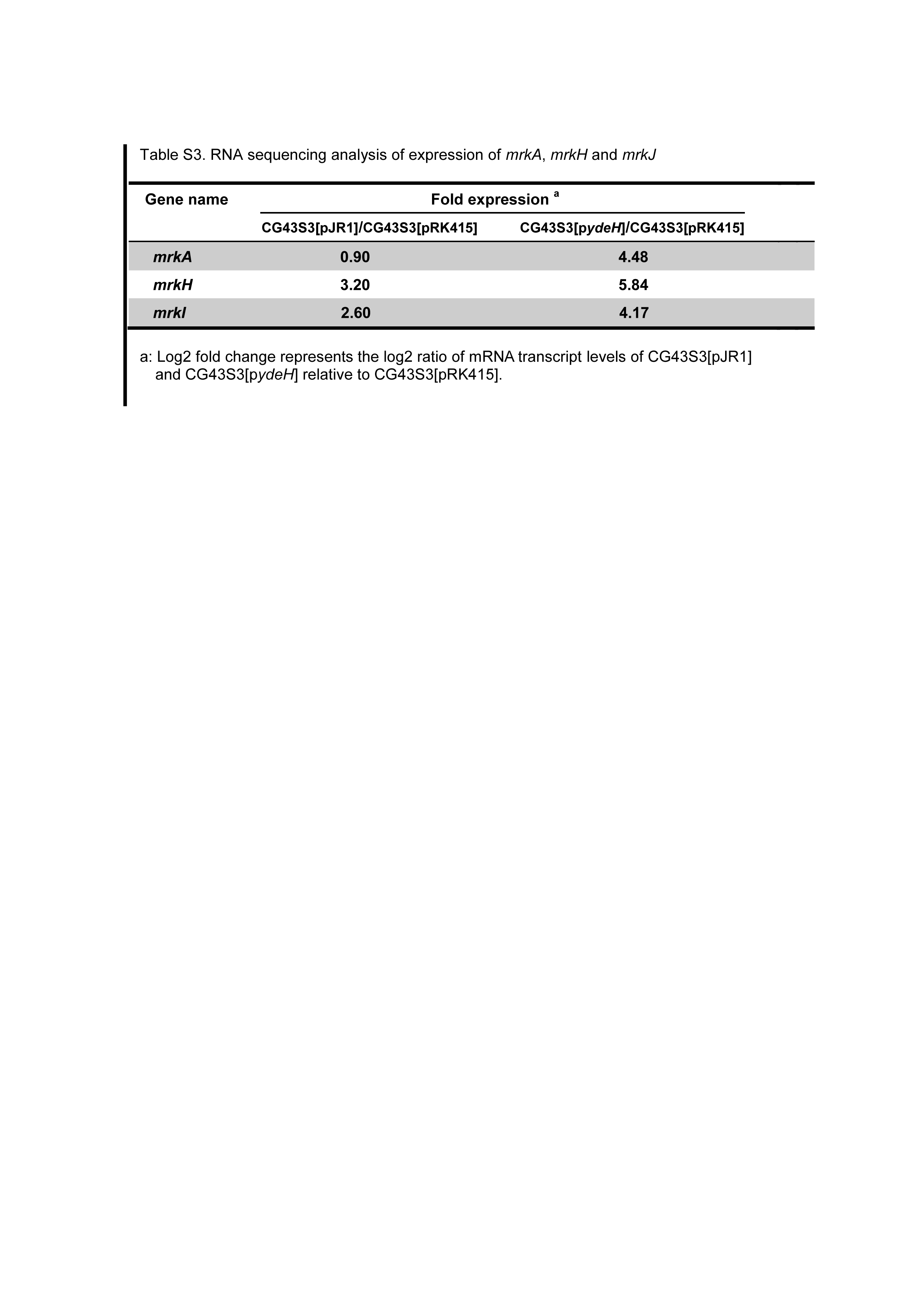

Supplement: Table S3 — RNA sequencing analysis of expression of mrkA , mrkH and mrkJ . (TIF) [file pone.0066740.s003.tif]
